# Comprehensive evaluation of extracellular small RNA isolation methods from serum in high throughput sequencing

**DOI:** 10.1186/s12864-016-3470-z

**Published:** 2017-01-07

**Authors:** Yan Guo, Kasey Vickers, Yanhua Xiong, Shilin Zhao, Quanhu Sheng, Pan Zhang, Wanding Zhou, Charles R. Flynn

**Affiliations:** 1 Department of Cancer Biology, Vanderbilt University, Nashville, TN USA; 2 Department of Medicine, Vanderbilt University Medical Center, Nashville, TN USA; 3 Department of Surgery, Vanderbilt University, MRBIV 8465A Langford Hall, 2213 Garland Ave, Nashville, TN 37232 USA; 4 Center for Epigenetics, Van Andel Research Institute, Grand Rapids, MI 49503 USA

## Abstract

**Background:**

DNA and RNA fractions from whole blood, serum and plasma are increasingly popular analytes that are currently under investigation for their utility in the diagnosis and staging of disease. Small non-coding ribonucleic acids (sRNAs), specifically microRNAs (miRNAs) and their variant isoforms (isomiRs), and transfer RNA (tRNA)-derived small RNAs (tDRs) comprise a repertoire of molecules particularly promising in this regard.

**Results:**

In this designed study, we compared the performance of various methods and kits for isolating circulating extracellular sRNAs (ex-sRNAs). ex-sRNAs from one healthy individual were isolated using five different isolation kits: Qiagen Circulating Nucleic Acid Kit, ThermoFisher Scientific Ambion TRIzol LS Reagent, Qiagen miRNEasy, QiaSymphony RNA extraction kit and the Exiqon MiRCURY RNA Isolation Kit. Each isolation method was repeated four times. A total of 20 small RNA sequencing (sRNAseq) libraries were constructed, sequenced and compared using a rigorous bioinformatics approach. The Circulating Nucleic Acid Kit had the greatest miRNA isolation variability, but had the lowest isolation variability for other RNA classes (isomiRs, tDRs, and other miscellaneous sRNAs (osRNA). However, the Circulating Nucleic Acid Kit consistently generated the fewest number of reads mapped to the genome, as compared to the best-performing method, Ambion TRIzol, which mapped 10% of the miRNAs, 7.2% of the tDRs and 23.1% of the osRNAs. The other methods performed intermediary, with QiaSymphony mapping 14% of the osRNAs, and miRNEasy mapping 4.6% of the tDRs and 2.9% of the miRNAs, achieving the second best kit performance rating overall.

**Conclusions:**

In summary, each isolation kit displayed different performance characteristics that could be construed as biased or advantageous, depending upon the downstream application and number of samples that require processing.

**Electronic supplementary material:**

The online version of this article (doi:10.1186/s12864-016-3470-z) contains supplementary material, which is available to authorized users.

## Background

Biomarkers come in many forms—proteins, nucleic acids, metabolites, small molecules—and can be evaluated as indicators of specific metabolic, physiologic, or pathologic states or conditions. Biomarkers have been used in numerous clinical assays to detect the presence or risk of developing disease. Biomarker assays should be conducted from an easily accessible source with minimally invasive medical procedures. One class of molecules, e.g., small RNAs (sRNAs), with great potential for biomarker utility is micro RNAs (miRNAs). miRNAs are 20-25nts in length and are one class of sRNAs that play vital roles in multiple cellular and developmental processes, primarily via post-transcriptional regulation of gene expression [1]. Currently, over 1880 annotated miRNAs (miRBase v21) have been reported in the human transcriptome, targeting >60% of coding genes in the genome [2]. These miRNAs are primarily transcribed by RNA polymerase II either as standalone transcription units or as part of the non-coding intronic sequence within a host gene. They typically function through interactions with Argonaute family proteins, which lead to the formation of a RNA-induced silencing complex (RISC) and suppressed gene expression [3]. At certain stages of the cell cycle, miRNAs have also been reported to assume an activating role in gene expression [4]. Mature miRNAs are also released from cells into circulation, and are therefore detectable in serum [5, 6], plasma [7] and all biological fluids tested. As such, extracellular RNA, particularly ex-sRNAs, have great potential as disease biomarkers in non-invasive assays.

miRNAs are becoming increasingly recognized for their potential to diagnose and stage disease, with cancer being a great example [8–13]. The utility of these miRNAs, in part, is due to their relatively high copy number, stable biochemical properties under clinical conditions, and discriminating transcription that can characterize unique physiological abnormalities. Despite the wide-range of studies that have been conducted to find sRNAs and disease associations, technical challenges continue to deter the utilization of sRNAs in clinical applications. One of the biggest issues for sRNA-based studies is the relatively low concentration of sRNAs present in serum and plasma samples. Currently, there are several miRNA extraction kits that are able to work with low input amounts and extract sRNAs from blood products. Previous sRNA studies [14–16] used a variety of extraction approaches, each with their own advantages and disadvantages. Yet, no consensus exists on the best approach.

Methods for RNA characterization can be classified into two major categories: hybridization-based microarray or synthesis/base-extension-based. Earlier sRNA studies mostly consisted of real-time quantitative polymerase chain reaction (RT-PCR) or hybridization-based assays. However, with the advancement of high-throughput sequencing technology, high-throughput sRNA screening has shifted from hybridization-based microarray technology to sRNAseq technology. One of the most considerable advantages that sRNAseq offers over microarrays is that it does not limit the detection of sRNA to a set of previously known targets. sRNAseq begins by constructing a cDNA sequence library reversely transcribed from short sRNA selected via different methods, e.g., size-selected gel electrophoresis. The prepared, indexed and pooled cDNA library can then be sequenced on different massive parallel sequencing platforms. Subsequent bioinformatic analysis of sRNAseq data provides the identification, quantification and differential expression of sRNAs. Since size-selection is agnostic to sRNA class (excluding potential chemical modifications), it has the potential to capture many species of sRNAs short in length, including miRNAs, miRNA isoforms (isomiRs) [17, 18], transfer RNA (tRNA)-derived small RNAs (tDRs) [19, 20], and other miscellaneous sRNAs (osRNAs) [21, 22]. IsomiRs are the isoforms of miRNA. The isomiRs usually have alternative seed sequences as compared to reference miRNA sequences [23]. The altered seed sequence can cause substantial differences in the repertoire of predicted mRNA targets. tDRs are the product of either active cleavage or an artifact of small RNA library construction. The parent tRNAs are adaptor molecules with a length typically ranging from 73 to 94 nucleotides. It is speculated that the cleavage of tRNAs by an RNAse III enzyme, angiogenin, may occur in a number of reactive conditions to produce tRNA-derived halves (tRHs) [24, 25]. The osRNAs we tried to detect include rRNA, snoRNAs, snRNA, lincRNA, and other miscellaneous sRNAs.

Although sRNA isolation and sRNAseq have primarily been used to quantify miRNAs, it is not completely understood as to what extent RNA isolation and sRNA library kits capture other types of sRNAs. Furthermore, there are many commercially available sRNA extraction kits, and the field would greatly benefit from a carefully designed study to evaluate and compare kit efficiency and reliability. Motivated by these reasons, we benchmarked five popular extraction kits for performance in aiding ex-sRNA analysis.

## Methods

### Reagents

Five miRNA extraction kits were obtained: Qiagen Circulating Nucleic Acid Kit (NAK), ThermoFisher Scientific Ambion TRIzol LS Reagent, Qiagen miRNEasy, QiaSymphony RNA extraction kit and the Exiqon MiRCURY RNA Isolation Kit. Highly pure diethyl pyrocarbonate (DEPC)-free and nuclease-free water were purchased from Qiagen.

### Sample handling

After obtaining informed consent, blood from a single subject was collected in pre-chilled tubes containing ethylenediamine-tetra-acetate and placed on ice. Samples were centrifuged at 3,000 x g for 10 min, aliquoted and stored at -80 °C until further analyses were performed. sRNAs from the serum of a single subject were extracted four times per kit. For each replicate, 200 μl of serum was used. RNA was extracted with the miRNEasy Serum/Plasma Kit, QIAamp Circulating NAK, miRCURY RNA Isolation—BioFluids Kit, and with Ambion TRIzol LS reagent according to manufacturer’s instructions. RNA was extracted on the QIAsymphony SP (Qiagen Corporation, Germany) using the QIAsymphony RNA Kit (Qiagen, 931636) and protocol RNA_CT_400_V7, which incorporates DNase treatment. The resulting RNA was eluted with RNase free water and stored at −80 °C until use. Samples were initially quantified using a Qubit fluorometric RNA assay (Life Technologies, Grand Island, NY). Additional analyses of purity and total RNA quantification were performed using a NanoDrop spectrophotometer (Thermo Scientific) and Agilent RNA 6000 Pico chip (Agilent) according to the manufacturer’s protocol using the reagents, chips, and ladder provided in the kit. The RNA concentration were measured using Qubit. However, since the targets are extracellular miRNA, the concentration were often below the detection threshold. Additionally, we verified the RNA concentration using Agilent Bioanalyzer. The concentrations are reported in Additional file 1: Table S1.

### Next-generation small RNA sequencing

RNAseq was performed by the Vanderbilt Technologies for Advanced Genomics core (VANTAGE). Libraries were prepared using the TruSeq Small RNA sample preparation kit (Illumina, San Diego, CA). The sRNA protocol specifically ligates RNA adapters to mature miRNAs harboring a 5’-phosphate and 3’-hydroxyl group as a result of enzymatic cleavage by RNase III processing enzymes, e.g., Dicer. In the first step, RNA adapters were ligated onto each end of the sRNA, and reverse transcription was used to create single-stranded cDNA. This cDNA was then PCR amplified for 18 cycles with a universal primer and a second primer containing one of 20 uniquely indexed tags to allow multiplexing. Size-selection of the cDNA constructs was performed using a 3% gel cassette on the Pippin Prep (Sage Sciences, Beverly, MA) to include only mature miRNAs and other sRNAs in the 5–40 bp size range and to remove adapter-adapter products. The resulting cDNA libraries then underwent a quality check on the Agilent Bioanalyzer HS DNA assay (Agilent, Santa Clara, CA) to confirm the final library size and on the Agilent Mx3005P quantitative PCR machine using the KAPA library quantification kit (Illumina, San Diego, CA) to determine concentration. A 2 nM stock was created, and samples were pooled by molarity for equimolar multiplexing. From the pool, 10 pM of the pool was loaded into each well of the flow cell on the Illumina cBot for cluster generation. The flow cell was then loaded and sequenced on the Illumina NextSeq500 to obtain at least 15 million single end (1x50 bp) reads per sample. The raw sequencing reads in BCL format were processed through CASAVA-1.8.2 for FASTQ conversion and de-multiplexed. The RTA chastity filter was applied, and only PF (pass filter) reads were retained for further analysis.

### Bioinformatics and data analyses

We implemented a custom in-house data analysis pipeline [19] for sRNAseq data processing. We categorized ex-sRNAs into four major categories: miRNAs, isomiRs, tDRs, and osRNAs. Cutadapt [26] was used to trim 3’ adapters for raw reads. Multi-perspective quality control [27] on raw data was performed using QC3 [28]. All reads with lengths less than 16nts in length were discarded. The adaptor-trimmed reads were formatted into a non-redundant FASTQ file, where the read sequence and copy number was recorded for each unique tag. The usable unique reads were mapped to the whole genome by Bowtie1 [29] allowing only one mismatch. In addition, our pipeline takes into consideration non-templated nucleotide additions [30–33] at the 3’ end of miRNAs during alignment, resulting in a more accurate miRNA expression quantification. The miRNA coordinates were extracted from miRBase. The tRNA (tDR) coordinates were prepared by combining the latest UCSC tRNA database GtRNAdb [34] with the tRNA loci of mitochondria from the Ensembl database [35]. The osRNA coordinates were extracted from the Ensembl database. The tDR reads were used not only for tDR quantification, but also for tRNA mapping position coverage analysis. ex-sRNAs were divided into three major categories: miRNAs, tDRs, and osRNAs (including sRNAs-derived from parent long non-coding RNAs (lncRNAs), snoRNAs, snRNAs, and miscellaneous RNAs in the Ensembl database). IsomiRs were detected by matching alignment of the reads at +1 or +2 positions from the start of the 5’ annotation of miRNAs.

The map and cluster analysis were performed using Heatmap3 [36] to evaluate the relationship between repeated samples based on the sRNA reads aligned. Furthermore, we computed intra-class correlation (ICC), a statistical measure of the homogeneity between more than two groups [37]. ICC (R package “irr”) was used to assess each kit’s agreement for sRNA expression measured from the replicates. ICC is a numerical value ranging from 0 to 1, where a higher value indicates more agreement among repeats. Additionally, we used Levene’s test [38] (R lawstat package) to assess the quality of variances for the sRNAs detected among repeats. Each kit was also evaluated based on the number of sRNA detected.

## Results

The yields of small RNA were measured by Agilent Bioanalyzer and varied greatly (by at least one order of magnitude) (Table S1). We computed the coefficients of variance (CV) of yields within each kit as assessments of the repeatability of yields. MiRCURY kit achieved the lowest CV of 0.04, followed by Ambio TRIzol (CV = 0.29), QiaSymphony (CV = 0.31), Circulating NAK (CV = 1.05) and miRNAEasy had the highest CV of 1.31.

**Fig. 1:**
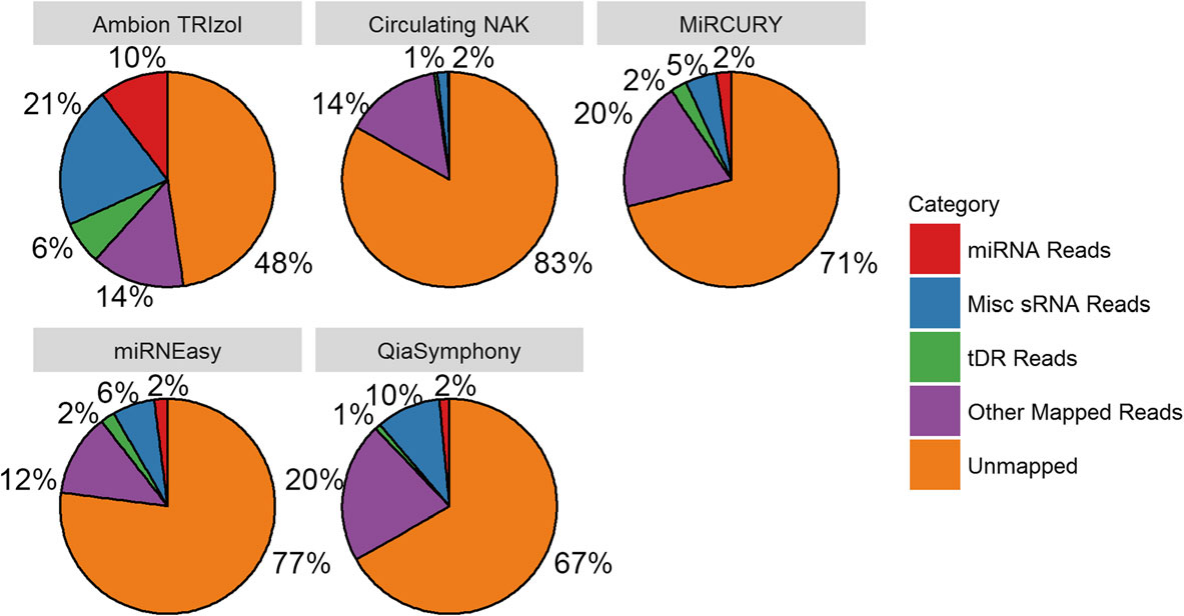
Pie chart that depicts the percentage of number of reads in different alignment categories: miRNA (include isomiR), tDR, osRNA, other mapped reads, unmapped. For any sRNA sequencing project on tissue, the unmapped rate will be at least 50%. The unmapped rate will be even higher for extracellular sRNA sequencing because low sRNA content in serum

The performance of each kit was first evaluated for sequencing quality and aligned reads. The factors we considered included: total number of reads sequenced, number of reads aligned to each of the sRNA classes, variation in number of reads, multiple alignment issues (reads perfectly mapped to multiple) genomic locations, and unmapped reads (Fig. 1, Additional file 1: Table S2). In regards to the total number of reads sequenced, Circulating NAK produced the most reads with an average of 32.9 million over the four replicates. However, the majority of the reads (83%) in samples isolated by the Circulating NAK were not mapped to the human genome. In contrast, QiaSymphony produced the fewest number of reads with 6.6 million; yet, 37% were mapped to the human genome. Ambion TRIzol yielded 11 million reads with 54% mapping, which was the highest of any method tested. All kits had at least 50% of reads not mapped to human genome which suggested that these reads were not RNA reads. For reads mapped to sRNAs, Ambion TRIzol consistently produced the most reads for miRNAs (23.1%), tDRs (7.2%), and osRNAs (23.1%). Circulating NAK performed poorly for all sRNAs species. Because equal amounts of the sequencing library from each replicate were pooled onto the same lane of Illumina NextSeq500, ideally, the number of reads sequenced for each sample should be roughly equal. However, variability in sequencing depth can be caused by many factors, including sample quality and statistical variation [39]. Since we used 20 technical replicates of the same sample, the observed variation likely reflects the efficiency and native difference of the kits rather than the sample quality. The completed read count table for the four types of sRNA are provided as supplementary data (Additional file 1: Table S3-S6).

**Fig. 2:**
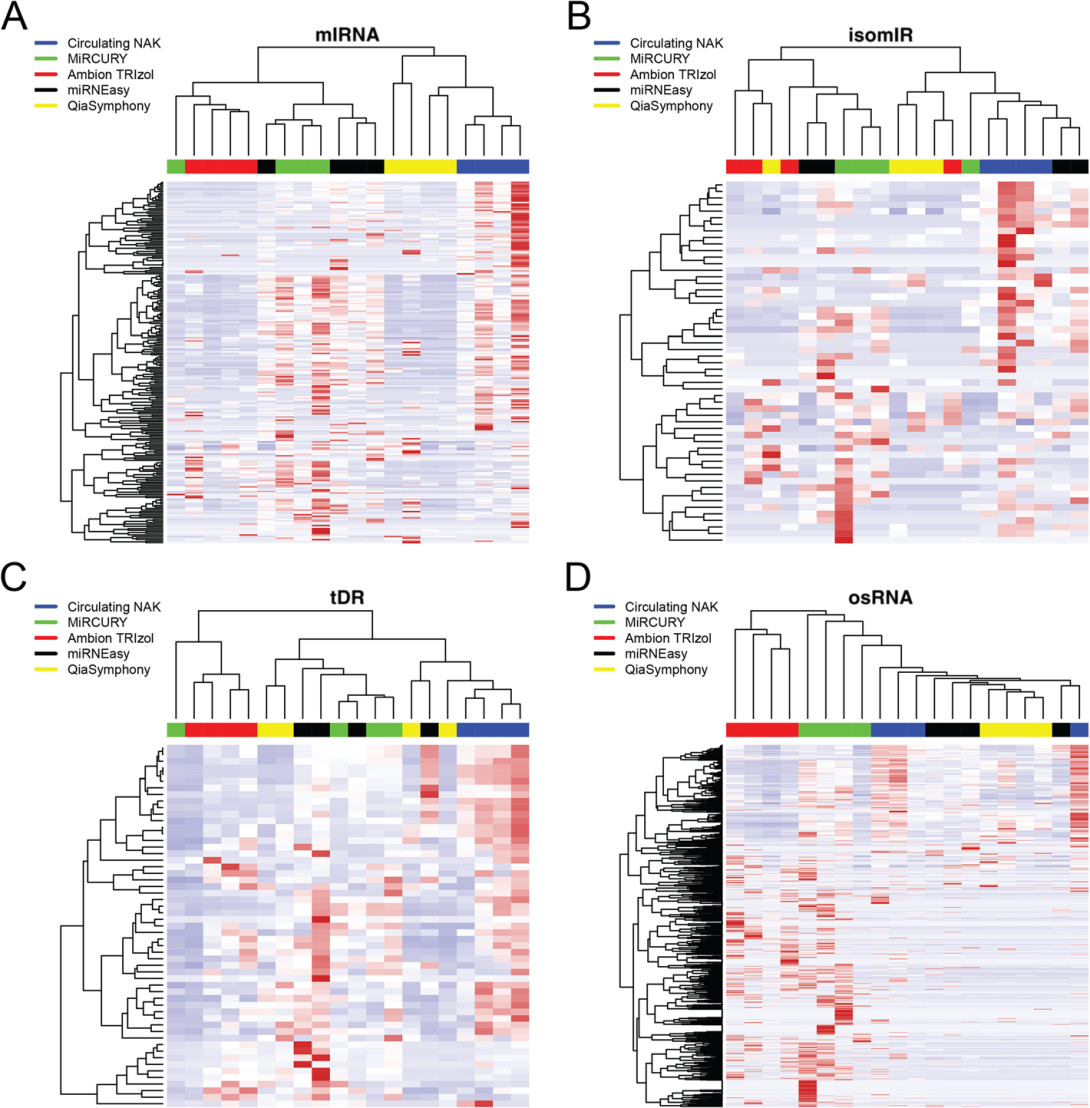
**a** Cluster and heatmap results for miRNA. **b** Cluster and heatmap results for isomiRs. **c** Cluster and heatmap results for detected tRNAs. **d** Cluster and heatmap results for miscellaneous sRNAs

We conducted unbiased cluster analysis using Heatmap3 [40] for each of the four major sRNA categories (Fig. 2). The cluster analysis essentially measures the repeatability of each kit, and inherent differences among the kits will cause some variability in the sRNA data. In contrast, replicate analyses of the same kit are expected to perform similarly and tightly cluster. For miRNAs, replicates using Ambion TRIzol, QiaSymphony and Circulating NAK clustered together; for isomiRs, replicates using Circulating NAK clustered together; for tDRs, replicates using Ambion TRIzol and Circulating NAK clustered together; for osRNA, Ambion TRIzol, miRCURY, and QiaSymphony clustered together. Overall, Ambion TRIzol and Circulating NAK produced the best cluster results for replicates.

**Fig. 3:**
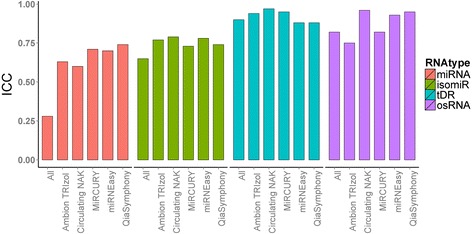
Intra-class correlation coefficient ranges from 0 to 1. Higher number indicates more agreement among replicates

We used ICC within each kit (based on four repeats per kit) to measure the repeatability of the extraction kits. For miRNAs, QiaSymphony achieved the highest ICC of 0.74; for isomiRs, tDRs, and osRNA, Circulating NAK achieved the highest ICCs of 0.79, 0.97 and 0.96, respectively. We also computed the ICC across all 20 samples to capture the overall homogeneity. It is worth noting that miRNAs had the worst overall ICC of 0.28, followed by isomiRs and osRNA. The mean ICC for tDRs across all isolated methods was 0.9 (Fig. 3, Additional file 1: Table S7). Using Levene’s test we found no significant difference in the equality of variances for the sRNAs detected among repeats (p = 1).

**Fig. 4:**
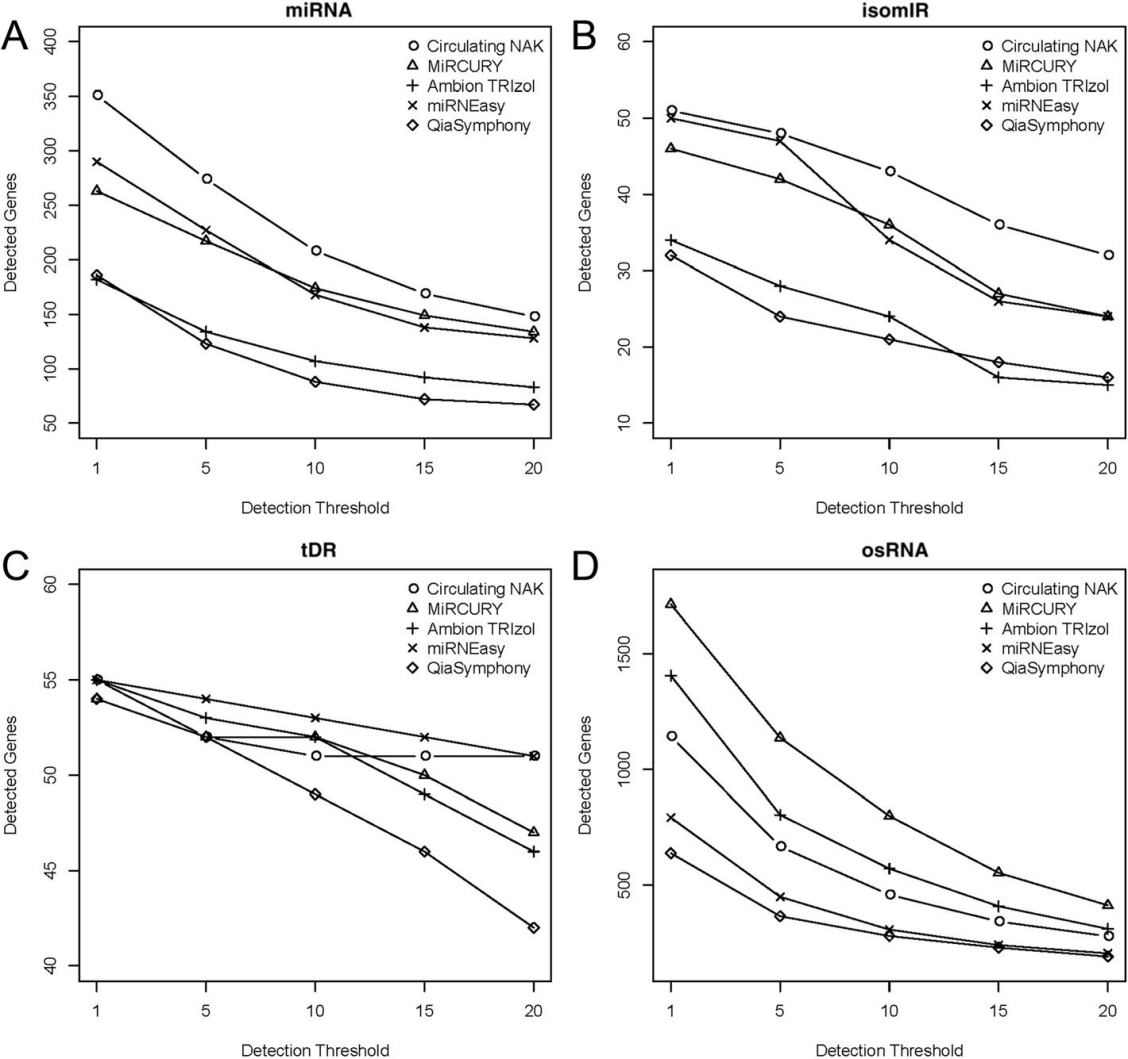
**a** Number of detected miRNA. **b** Number of detected isomiR. **c** Number of detected tRNA. **d** Number of detected miscellaneous RNA. X-axis denotes the read count threshold used for detection. As the detection threshold increased, less RNAs were identified

**Fig. 5:**
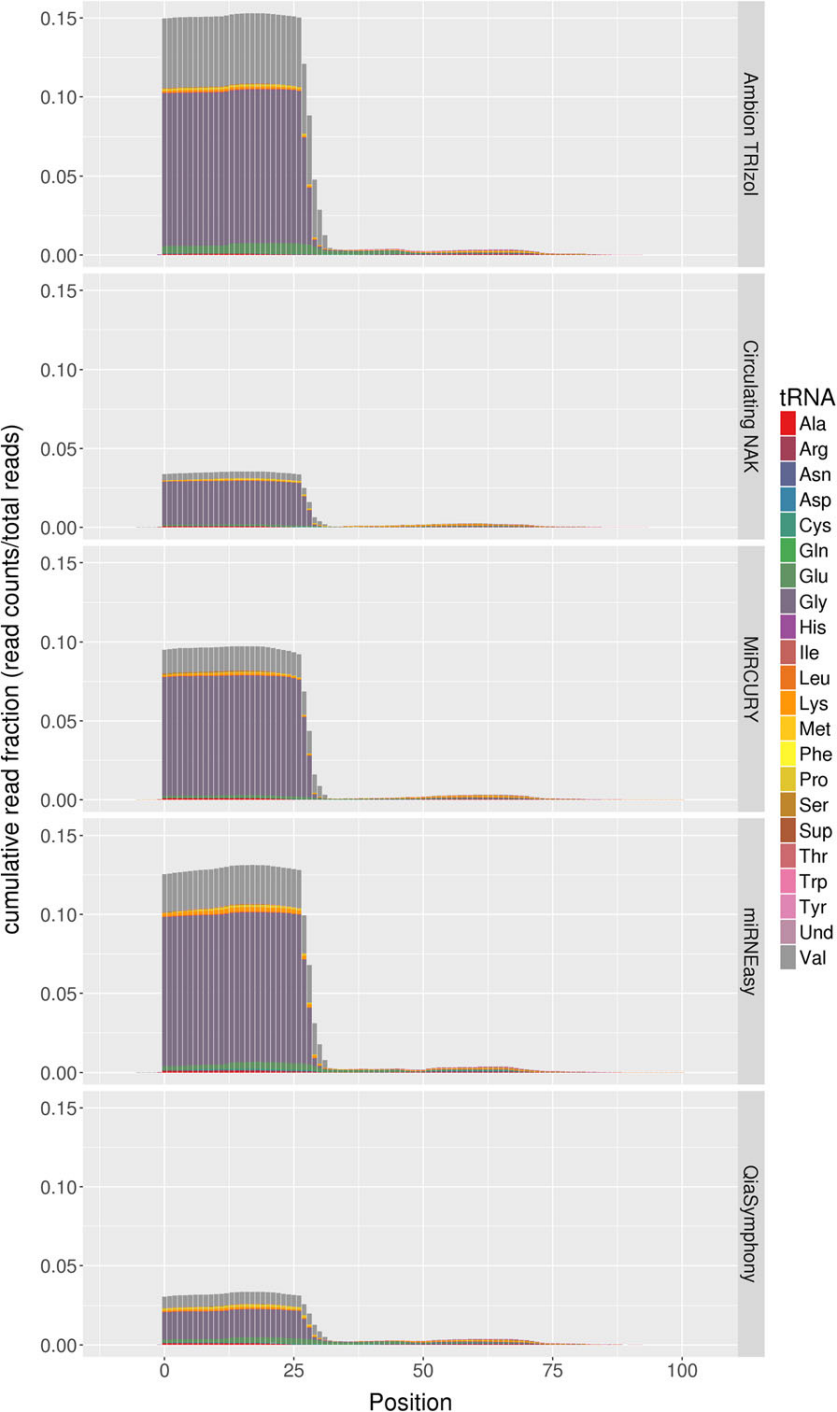
tRNA positional alignment distribution. Color indicates the tRNA by anticodon type. The x-axis denotes the position of tRNA from 0 to the end of tRNA. The y-axis denotes the cumulative read fractions. Visible quantity difference can be seen in the tRNA type detected by different kits. This suggests that there are selection bias of tRNA in by the kits

Next, we examined the number of detected sRNAs by each kit (Fig. 4). To determine if a sRNA was detected, we selected several detection thresholds which have been commonly used for assessing sRNA detection (read counts >1, >5, >10, >15, >20) [41]. Circulating NAK consistently detected the greatest number of miRNAs and isomiRs at all detection thresholds. For tDRs, the miRNEasy kit detected the most sRNAs at all thresholds, but Circulating NAK also performed equally well for the lowest and highest detection thresholds. For osRNAs, the miRCURY kit performed the best at all detection thresholds. We also performed the tRNA alignment pattern analysis (5’ end on the left, 3’ end on the right), color coded by anticodon type, which showed some difference in alignment patterns of tDRs among the kits (Fig. 5). For Ambion TRIzol and miRNEasy, a higher percentage of the reads were aligned to parent tRNAs, and Circulating NAK and QiaSymphony had the lowest percentage of reads aligned to parent tRNAs. The read distributions by anticodon type of tRNA were also different among the kits.

**Fig. 6:**
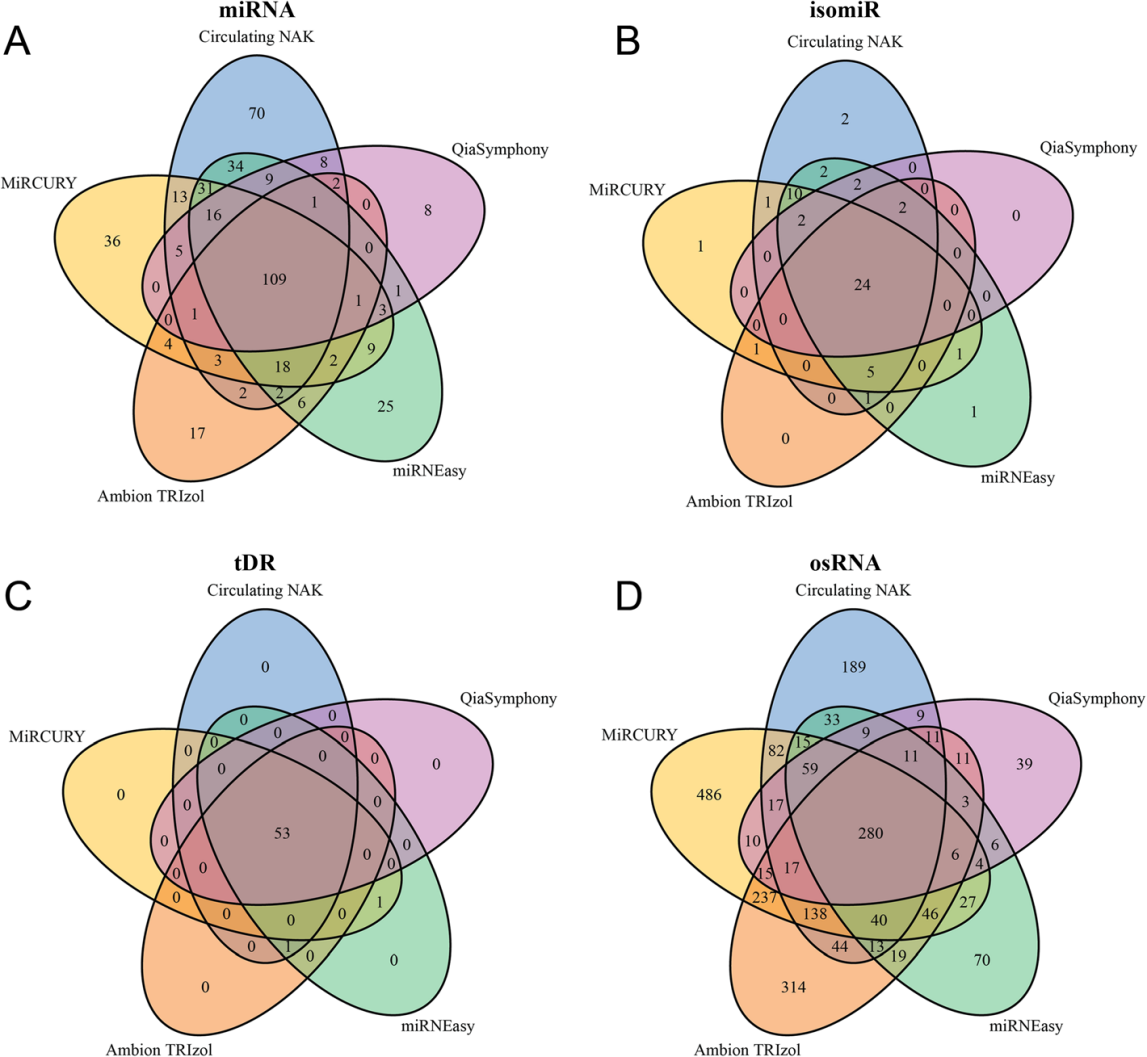
Venn diagrams to represent the intersection and uniquely detected sRNAs, detection threshold used was read count > 10. **a** The intersection of detected miRNA by all kits. **b** The intersection of detected isomiRs by all kits. **c** The intersection of detected tRNA by all kits. **d** The intersection of detected miscellaneous sRNA by all kits

However, the kit that detects the most sRNAs might also detect the most singleton miRNAs. We define a singleton as a species that is detected by one of the five isolation kits. Circulating NAK detected the most singleton miRNAs, which explains why Circulating NAK also detected the most miRNAs. Very few singleton isomiRs and no singleton tDRs were detected. miRCURY detected the most singleton osRNAs (Fig. 6). A list of the top singleton miRNAs, computed and ranked by the differences between one kit and the other four kits, is available in the Additional file 1: Table S8. Using Levene’s test, we found that the commonly detected sRNAs have no significant amount of difference in level of expression among kits. The presence of singleton sRNA may represent each kit’s uniqueness and can be interpreted as either advantageous or biased. A list of the top other sRNAs, computed and ranked by the differences between one kit and the other four kits, is available in the Additional file 1: Table S9.

## Discussion

ex-sRNAs have the potential to make a strong impact in the field of biomarker research. The ability to detect ex-sRNA from easily obtainable medium, such as serum or liquid biopsies, can greatly enhance the capability of molecular diagnose [42–44]. A reproducible biomarker also provides valuable insights into specific biological traits at the molecular and cellular level. While having great potential, the analysis of ex-sRNAs from high throughput sequencing pose serious bioinformatics challenges [45]. Currently, one of the difficulties preventing the full fruition of ex-sRNA as a reliable biomarker analysis is the consistency of detection. Another potential issue clouding the future of ex-sRNAs is the obscurity of the origin of these nucleic acids in human serum/plasma. Some arguments have been made that ex-miRNAs can be released into the blood stream directly from blood cells [46] and/or other tissue cells [47].

Our study was not designed to study the origin of sRNAs detected in serum. Instead, our study was motivated by the lack of consensus over the best approach for ex-sRNA isolation. To tackle this problem, we designed a thorough experiment to evaluate the performance of five sRNA isolation kits that have been previously used for isolating ex-miRNAs. Through careful examination of the sequencing data, we can safely conclude that not only were ex-miRNAs detectable in serum, but other species of sRNA, including, ex- isomiRs, ex-tDRs, and ex-osRNAs, were also detectable. We primarily examined the performance of the isolation kits from three aspects: 1) the number of reads sequenced and aligned to each species of sRNA; 2) the repeatability of the replicates within each kit; 3) the number of sRNAs detected. While each kit was used per manufacturer’s instructions, and the amount of nucleic acid input into each replicate for each kit was the same, there were notable kit-specific differences in RNA yield. Each kit designated a different volume of water for elution which is reflected in the concentration and total amount of RNA yielded, and may impart a certain level of bias in our results.

All five kits had poor mapping rate to human genome (<50%), suggesting all kits captured a large amount of miscellaneous material. The phenomenon of high unmapped rates is quite common for high throughput sequencing experiments, however the scale of the unmapped rate depends on the sample quality, type of sequencing, and the abundance of the source RNA. In exome sequencing, it had been shown that the unmapped rate is anywhere from 5 to 19% [48]. For small RNA sequencing on tissue samples, we have previously reported the unmapped rate between 30 and 40% [20]. Our current study focused on ex-sRNAs from serum which is of much less abundance compared to tissue samples. The high unmapped rate is a reflection of the nature of the experiment.

Ambion TRIzol had the most reads annotated as sRNAs; however, the observed increase in genomic alignments did not translate into a higher number of detected sRNAs, suggesting either there was selection bias for Ambion TRIzol or the reads were dominated by miRNAs with high expression. Reproducibility was evaluated using cluster analysis and ICC, and Circulating NAK had the highest repeatability overall. In terms of the number of ex-sRNAs detected, Circulating NAK detected the most ex-osRNAs, ex-isomiRs and also performed well for ex-tDRs. miRCURY detected the highest number of ex-osRNAs. However, currently, ex-osRNAs are the least studied sRNAs and their biological functions remain largely unknown.

In sRNA sequencing, especially extracellular sequencing, the detection threshold of miRNA can significantly affect the number of sRNAs detected. In our study, for demonstration purposes, we have used detection threshold of read counts >1, >5, >10, >15, >20. In reality, sRNA detected with less than 10 read counts are difficult to replicate by RT-PCR. For reliable sRNA detection, it is recommended to set a detection threshold with read count > 10 [49].

## Conclusions

In conclusion, our data suggest that each isolation kit displays inherent performance characteristics that may be construed as biased, or advantageous, depending upon the downstream application and number of samples that require processing. The Circulating NAK consistently generated the fewest number of reads mapped to the genome, in comparison to the best performing method, Ambion TRIzol, where 10% of the detected miRNAs, 7.2% of the tDRs and 23.1% of osRNAs were mapped. The performance of the other methods was intermediary, with QiaSymphony mapping 14% of osRNAs and miRNEasy mapping 4.6% of tDRs and 2.9% of miRNAs, making it the second best performing kit in terms of sRNA extraction efficiency. However, the Circulating NAK kit detected the highest number of miRNAs. These data suggest that the choice of sRNA isolation kits for ex-sRNA analysis is not trivial and may introduce significant bias that must be addressed when interpreting outcomes.

## Additional file

Additional file 1: Supplementary Tables S1-S9. (XLSX 576 kb)

## Abbreviations

ex: extracellular
isomiR: Micro RNA isoform
lncRNA: long non-coding RNAs
miRNA: micro RNA
NAK: Nucleic acid kit
RT-PCR: Quantitative polymerase chain reaction
sRNA: small RNA
sRNAseq: small RNA sequencing
tDR: transfer RNA derived small RNA
tDR: transfer RNA derived small RNAs
tRNA: transfer RNA
